# Plasmalogens Inhibit APP Processing by Directly Affecting **γ**-Secretase Activity in Alzheimer's Disease

**DOI:** 10.1100/2012/141240

**Published:** 2012-04-01

**Authors:** Tatjana L. Rothhaar, Sven Grösgen, Viola J. Haupenthal, Verena K. Burg, Benjamin Hundsdörfer, Janine Mett, Matthias Riemenschneider, Heike S. Grimm, Tobias Hartmann, Marcus O. W. Grimm

**Affiliations:** ^1^Experimental Neurology, Saarland University, Kirrbergerstra*β*e, 66421 Homburg/Saar, Germany; ^2^Psychiatry, Saarland University, Kirrbergerstra*β*e, 66421 Homburg/Saar, Germany; ^3^Deutsches Institut für DemenzPrävention (DIDP), Universität des Saarlandes, Kirrbergerstra*β*e, 66421 Homburg/Saar, Germany; ^4^Neurodegeneration and Neurobiology, Saarland University, Kirrbergerstra*β*e, 66421 Homburg/Saar, Germany

## Abstract

Lipids play an important role as risk or protective factors in Alzheimer's disease (AD). Previously it has been shown that plasmalogens, the major brain phospholipids, are altered in AD. However, it remained unclear whether plasmalogens themselves are able to modulate amyloid precursor protein (APP) processing or if the reduced plasmalogen level is a consequence of AD. Here we identify the plasmalogens which are altered in human AD *postmortem* brains and investigate their impact on APP processing resulting in A*β* production. All tested plasmalogen species showed a reduction in *γ*-secretase activity whereas *β*- and *α*-secretase activity mainly remained unchanged. Plasmalogens directly affected *γ*-secretase activity, protein and RNA level of the secretases were unaffected, pointing towards a direct influence of plasmalogens on *γ*-secretase activity. Plasmalogens were also able to decrease *γ*-secretase activity in human *postmortem* AD brains emphasizing the impact of plasmalogens in AD. In summary our findings show that decreased plasmalogen levels are not only a consequence of AD but that plasmalogens also decrease APP processing by directly affecting *γ*-secretase activity, resulting in a vicious cycle: A*β* reduces plasmalogen levels and reduced plasmalogen levels directly increase *γ*-secretase activity leading to an even stronger production of A*β* peptides.

## 1. Introduction

**Figure 1:**
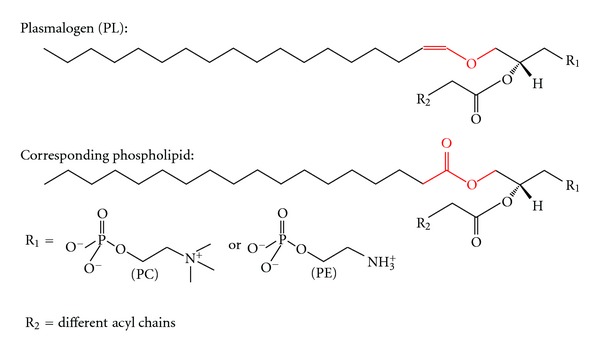
Structure of plasmalogen (PL) and the corresponding phospholipid used in this study. In the plasmalogens, the fatty acid is linked via an enol ether bond instead of an ester bond marked in red. Residue 1 (R1) can either be a phosphatidylcholine or a phosphatidylethanolamine leading to PC-plasmalogen or PE-plasmalogen. The sn-2 position can vary in different fatty acids symbolized by residue 2 (R2).

Plasmalogens are glycerophospholipids and major constituents of neuronal membranes. Beside human brain, where plasmalogens represent almost 20% of total glycerophospholipids, they can be found in all mammalian tissues, especially in the heart muscle [1–3]. Characteristic of plasmalogens is an enol ether double bond at the sn-1 position of the glycerol backbone (Figure 1), which makes plasmalogens more susceptible to oxidative stress than the corresponding ester-bonded glycerophospholipid, thus protecting cells from oxidative stress [4]. Beside their function as antioxidants, plasmalogens are involved in membrane fusion [5, 6], ion transport [7–9], and cholesterol efflux [10, 11]. Furthermore, plasmalogens can be hydrolyzed by plasmalogen-selective phospholipase A2 [3, 12], generating fatty acids like arachidonic acid, which is important for modulating ion channels, regulating different enzyme activities like protein kinase A, protein kinase C, NADPH oxidase, Na^+^K^+^-ATPase, and others [13]. Arachidonic acid released from plasmalogens can be metabolized to eicosanoids, acting as second messengers [14]. Due to the fact that plasmalogens represent major constituents of neuronal membranes and are involved in different cellular processes, it is not unexpected that neuronal function also depends on a delicate balance in lipid composition of cellular membranes. Alterations of plasmalogen levels occur in several neurological disorders including Alzheimer's disease (AD) [15–17], spinal cord trauma [18], ischemia [19, 20], Niemann-Pick disease [21], and multiple sclerosis [22]. For AD, plasmalogen levels have been described to be reduced in autopsy brain samples from AD patients compared to age-matched control brains [15–17, 23, 24]. However, Pettegrew et al. reported no differences or even a slight increase in AD patients [25]. One of the characteristic pathological hallmarks of AD is the massive accumulation of a small peptide, called amyloid beta peptide (A*β*) that aggregates in amyloid plaques [26, 27]. A*β* is generated by sequential processing of the amyloid precursor protein (APP), a type I integral membrane protein [28]. For the generation of A*β*, APP is first cleaved by *β*-secretase BACE1, a membrane-bound aspartyl-protease [29], generating *β*-secreted APP (sAPP*β*), and a C-terminal membrane-bound fragment, called C99 or *β*-CTF. C99 is further processed by *γ*-secretase, releasing the A*β* peptide. The *γ*-secretase has been identified as a multimeric complex of at least four transmembrane proteins, presenilin 1 (PS1) or presenilin 2 (PS2), nicastrin, anterior pharynx-defective 1 (Aph-1), and presenilin enhancer 2 (Pen-2) [30]. The polytopic transmembrane proteins PS1 or PS2 constitute the active site of the protease [31]. Beside the amyloidogenic processing of APP involving *β*- and *γ*-secretase activity, APP can be cleaved in a nonamyloidogenic pathway by *α*-secretases [32, 33]. The *α*-secretases have been identified as members of the ADAM family (a disintegrin and metalloproteinase), cleaving APP within the A*β* domain and therefore prevent the formation of A*β* [33–35]. As APP and its processing secretases are all integral membrane proteins, we analyzed in this study whether plasmalogens, major components of neuronal membranes, influence amyloidogenic and nonamyloidogenic processing of APP.

## 2. Materials and Methods

### 2.1. Chemicals and Reagents

All phosphatidylcholine and phosphatidylethanolamine species used in this study were purchased from Avanti Polar Lipids (Alabaster, AL, USA). Bovine serum albumin was purchased from Roth (Karlsruhe, Germany). All other reagents if not otherwise stated were purchased from Sigma Aldrich (Taufkirchen, Germany).

### 2.2. Cell Culture

SH-SY5Y cells were cultivated in Dulbecco's Modified Eagle's Medium (Sigma, Taufkirchen, Germany) with 10% FCS (PAN Biotech, Aidenbach, Germany). For incubation phospholipids solved in ethanol p.a. (Sigma, Taufkirchen, Germany) were added in a final concentration of 100 *μ*M to culture media with 0.1% FCS. Incubation was carried out for 24 h with changing incubation medium with phospholipids after 12 h. Lactate Dehydrogenase-assay analysis revealed no signs for elevated cytotoxicity or reduced membrane integrity in presence of phospholipids (which is available at doi:10.1100/2012/141240).

### 2.3. Brain Samples

In total, 58 human *postmortem* brain samples from 21 control and 37 Alzheimer's disease patients were used. For more details, see Table 1. Furthermore, for *ex vivo* analysis of *γ*-secretase activity postnuclear fractions from further 6 human *postmortem* brains obtained from confirmed AD patients were utilized. All human *postmortem* brains were obtained from BrainNet (Munich, Germany). In addition, postnuclear fractions from C57BI6/N wild-type mice were used. Preparation of postnuclear fractions is described in detail below.

### 2.4. Protein Amount Determination

All samples, including human *postmortem* brains and cells, were homogenized on ice using a PotterS (Braun, Melsungen, Germany) at 1500 revolutions per minute and 50 strokes. Protein determination was carried out according to Smith et al. [37]. Briefly, 20 *μ*L of bovine serum albumin in a concentration range of 0.1–1.2 *μ*g/*μ*L were used for determination of the standard curve. For determination of samples' protein amount, 1-2 *μ*L of each sample was loaded in triplicate onto a 96 well plate (Nunc, Langenselbold, Germany). 200 *μ*L of reagent buffer (CuSO_4_ : bicinchoninic acid; 1 : 39; Sigma Aldrich, Taufkirchen, Germany) was added to each well using a multichannel pipette (Eppendorf, Hamburg, Germany). Incubation took place for 15 minutes at 37°C and afterwards for further 15 minutes at room temperature while shaking (IKA, Staufen, Germany) at 300 revolutions per minute. Absorbance was measured at a wavelength of 560 nm using a MultiscanEX (Thermo Fisher Scientific, Schwerte, Germany).

### 2.5. Western Blot Analysis

For detection of ADAM17, PS1 and BACE1 protein amount, proteins of cell lysate were separated on 10%–20% Tricine gels (Anamed, Groß-Bieberau, Germany). Western Blot (WB) analysis was performed using antibody ab39162 (1 : 5000; abcam, Cambridge, UK), sc-7860 (1 : 500; Santa Cruz, Heidelberg, Germany), and B0806 (1 : 1000; Sigma, Taufkirchen, Germany) respectively. W401B (1 : 10000; Promega, Mannheim, Germany) was used as secondary antibody, and detection was carried out using Western Lightning Plus-ECL solution (Perkin Elmer, Rodgau, Germany). Densiometric quantification was performed using Image Gauge software.

### 2.6. Postnuclear Fractions

For preparing postnuclear fractions (PNFs) SH-SY5Y wild-type cells, mouse brains or human AD brains were washed with PBS and homogenized in sucrose-buffer (pH 7.4) using a PotterS (Braun, Melsungen, Germany) at maximum speed. Protein amount was adjusted to 2 mg/mL for *β*- and *γ*-secretase assay and to 1 mg/mL for measuring *α*-secretase activity. After centrifugation at 900 rcf for 10 min at 4°C supernatants were collected and stored at −80°C.

### 2.7. *In Vitro* Incubation

PNFs were warmed up at 37°C, and phospholipids solved in ethanol p.a. were added in a final concentration of 100 *μ*M. Samples were incubated while shaking (Multireax, Heidolph Instruments, Schwabach, Germany) for 15 min at 37°C before being centrifuged at 55.000 rpm for 75 min at 4°C for pelleting membranes.

### 2.8. Secretase Activities

***α*-Secretase Activity**

Pelleted SH-SY5Y membranes were resuspended in 1 mL, and purified mouse brain membranes were resuspended in 2 mL Hepes-buffer pH 7.5. For solubilisation, samples were passed through needles (BD, Franklin Lakes, NJ, USA) with decreasing diameters. Samples were dispended in triplicate onto a black 96-well plate using 100 *μ*L per well corresponding to 100 *μ*g for SH-SY5Y and 50 *μ*g for mouse brain membranes. After adding 3 *μ*M *α*-secretase-substrate (Calbiochem, Darmstadt, Germany), fluorescence was detected with excitation wavelength at 340 ± 10 nm and emission wavelength at 490 ± 10 nm using Safire^²^ Fluorometer (Tecan, Crailsheim, Germany). Kinetic was plotted for 120 cycles with kinetic intervals of 120 s. For specificity control, 10 mM EDTA/EGTA was used (supplement Figure S2).

***β*- and *γ*-Secretase Activities**

Pelleted membranes were resuspended in 800 *μ*L sucrose-buffer for SH-SY5Y membranes and 400 *μ*L for mouse brain and human AD brain membranes. Membranes were solubilized as described above. For analysing *γ*-secretase activity, samples were dispensed in triplicate on black 96-well plates (Corning, Lowell, MA, USA) using 100 *μ*L per well corresponding to 250 *μ*g for SH-SY5Y membranes and 500 *μ*g for mouse and human brain membranes. After adding 10 *μ*M *γ*-secretase substrate (Calbiochem, Darmstadt, Germany), fluorescence was measured with excitation wavelength 355 ± 10 nm and fluorescence detection at 440 ± 10 nm in a Safire^2^ Fluorometer (Tecan, Crailsheim, Germany) at 37°C under light exclusion. Kinetics were plotted for 50 cycles with kinetic intervals of 180 s. For determination of assay specificity, we used *γ*-secretase Inhibitor X (50 *μ*M) (Calbiochem, Darmstadt, Germany) (supplement Figure S3). For measuring *β*-secretase activity, samples were dispensed in triplicate on black 96 well plates (50 *μ*L per well corresponding to 125 *μ*g for SH-SY5Y membranes and 250 *μ*g for mouse brain membranes). *β*-secretase substrate (Calbiochem, Darmstadt, Germany) was added with a final concentration of 20 *μ*M and fluorescence was detected with an excitation wavelength at 345 ± 5 nm and emission wavelength at 500 ± 2.5 nm under light exclusion at 37°C. Kinetics were plotted for 180 cycles with kinetic intervals of 60 s. Assay specificity was validated using *β*-secretase Inhibitor II (1 *μ*M) (Calbiochem, Darmstadt, Germany) (supplement Figure S4). For all secretase assays, the unspecificity was between 10% to 30%. The secretase activities presented, were calculated by subtracting the unspecific turnover determined by adding secretase inhibitors.

### 2.9. Mass Spectrometry Analysis

For determination of phosphatidylcholine and phosphatidylethanolamine levels in human control and AD brains, we used a 4000 quadrupole linear-ion trap (QTrap) equipped with a Turbo-V ion source (AB SCIEX, Darmstadt, Germany) connected to a 1200 Agilent HPLC (Agilent, Böblingen, Germany). Briefly, samples were adjusted to 6 mg/mL protein amount and 10 *μ*L of the adjusted samples was pipetted onto a membrane (Whatman, GE Healthcare, Freiburg, Germany) fixed in the wells of a MultiScreen, solvinert 96-well plate with a 0.45 *μ*m sterile filter at the bottom (Millipore, Schwalbach, Germany). This 96 well plate was placed onto a 1 mL 96-well deep well plate (Nunc, Langenselbold, Germany). Samples were dried under a gentle flow of nitrogen for at least 30 min at room temperature. Meanwhile, phenylisothiocyanate was diluted in ethanol : water : pyridine (1 : 1 : 1; v/v/v) to obtain a final 5% phenylisothiocyanate solution. 20 *μ*L of this solution was pipetted onto each membrane, and the plate was incubated for 20 min at room temperature and afterwards dried under a gentle flow of nitrogen for at least 30 min. Samples were extracted using 300 *μ*L 5 mM ammonium acetate buffer in methanol using a multichannel pipette (Eppendorf, Germany) and the plate was shaken at 300 revolutions per minute using a plate shaker (IKA, Staufen, Germany) at room temperature for 30 min. Samples were centrifuged (Thermo Scientific, Langenselbold, Germany) at 500 ×*g* for 2 min through 0.45 *μ*m sterile filters into the 96 deep well plate and further diluted with 600 *μ*L of 5 mM ammonium acetate dissolved in methanol : water (97 : 3, v/v) which also was used as the only running solvent. Finally plate was covered with a silicone mat and shook for further 2 min at 300 rpm at room temperature. Plate was placed into the cooled autosampler and detection was carried out using Analyst 1.5 software (AB SCIEX, Darmstadt, Germany). Phosphatidylcholine species were detected using MRM transitions (PCae C34:1: 746,6 m/z − 184 m/z; PCae C36:4: 768,6 m/z − 184 m/z; PCae C36:2: 772,6 m/z − 184 m/z; PCae C36:1: 774,6 m/z − 184 m/z; PCae C38:6: 792,6 m/z − 184 m/z; PCae C38:5: 794,6 m/z − 184 m/z; PCae C38:4: 796,6 m/z − 184 m/z; PCae C40:6: 820,6 m/z − 184 m/z), and phosphatidylethanolamine species were detected using a neutral loss scan for 141 m/z (PEae 36:4: 728,8 m/z; PEae 36:2: 732,8 m/z; PEae 38:6: 752,8 m/z; PEae 38:5: 754,8 m/z; PEae 38:4: 756,8 m/z; PEae 40:6: 780,8 m/z). For both species detection, 20 *μ*L sample was injected into sample loop with the following running solvent gradient (0.0–2.4 min, 30 *μ*L; 2.4–3.0 min, 200 *μ*L; 3.0 min, 30 *μ*L).

### 2.10. Quantitative Real-Time Experiments

RNA was extracted in total from cells using TRIzol reagent (Invitrogen, Karlsruhe, Germany), according to manufacturer's protocols. High Capacity cDNA Reverse Transcription Kit (Applied Biosystems, Darmstadt, Germany) was used to reverse-transcribe 2 *μ*g total RNA. Quantitative real-time PCR analysis was carried out using Fast SYBR Green Master Mix on 7500 Fast Real-Time PCR System (7500 Fast System SDS Software 1.3.1.; Applied Biosystems, Darmstadt, Germany). All results were normalized to *β*-actin gene expression and changes detected in gene expression were calculated using 2^−(ΔΔCt)^ method [38]. The following primer sequences were used: *ADAM17: *5′-CTG TGT GCC CTA TGT CGA TG-3′ and 5′-CAG CTG GTC AAT GAA ATC CC-3′; *BACE1: *5′-GCA GGG CTA CTA CGT GGA GA-3′ and 5′-TAG TAG CGA TGC AGG AAG GG-3′; *PSEN1: *5′-CTC AAT TCT GAA TGC TGC CA-3′ and 5′-GGC ATG GAT GAC CTT ATA GCA-3′; *PSEN2: *5′-GAT CAG CGT CAT CGT GGT TA-3′ and 5′-GGA ACA GCA GCA TCA GTG AA-3′; *APH1a: *5′-CAG CCA TTA TCC TGC TCC AT-3′ and 5′-GGA ATG TCA GTC CCG ATG TC-3′; *APH1b: *5′-GTG TCA GCC CAG ACC TTC AT-3′ and 5′-CAG GCA GAG TTT CAG GCT TC-3′; *NCSTN*: 5′-CTG TAC GGA ACC AGG TGG AG-3′ and 5′-GAG AGG CTG GGA CTG ATT TG-3′; *PSENEN: *5′-CAT CTT CTG GTT CTT CCG AGA G-3′ and 5′-AGA AGA GGA AGC CCA CAG C-3′; **β*-Actin: *5′-CTT CCT GGG CAT GGA GTC-3′ and 5′-AGC ACT GTG TTG GCG TAC AG-3′. To verify the results obtained by quantitative real time experiments, samples were separated on 3% agarose gels in TBE buffer (90 mM Tris, 90 mM boric acid, 2 mM EDTA, pH8.0).

### 2.11. Cytotoxicity Measurement

Cytotoxicity was measured utilizing Lactate Dehydrogenase Cytotoxicity Assay Kit (Cayman Chemical, Ann Arbor, USA) according to manufacturer's protocol.

### 2.12. Statistical Analysis

All quantified data presented here is based on an average of at least three independent experiments. Error bars represent standard deviation of the mean. Statistical significance was determined by two-tailed Student's *t*-test; significance was set at *P ≤ 0.05; **P ≤ 0.01 and ***P ≤ 0.001.

## 3. Results

### 3.1. Determination of PC-PL and PE-PL Levels in Human AD Postmortem Brain

In our previous studies, we reported a decrease in total phosphatidylethanolamine-plasmalogen (PE-PL) [39] and some phosphatidylcholine-plasmalogen (PC-PL) species [40] in *postmortem* AD brains. To elucidate the question which plasmalogen species are mostly changed in AD, we determined in this study the major PE-PL and PC-PL species. In total, we analyzed 58 human brain samples, 21 control brains with an average age of 75 years, and 37 brain samples obtained from AD patients with an average age of 78 years (Table 1). There were no significant differences in the age and sex of AD patients and controls. Mass spectrometry analysis revealed that PC-PL levels were significantly reduced in AD *postmortem* brains compared to healthy individuals, whereas PC-PL 18 : 1/18 : 1 showed the strongest reduction to 49,17% (Table 2). PE-PL levels were also reduced; however, changes in individual species were not significant (Table 2). However, the question whether a reduced plasmalogen level is caused by AD or plasmalogens themselves affect AD by directly affecting APP processing remained unclear and is addressed by the following experiments. As PC-PL 18 : 1 showed the strongest reduction in AD *postmortem* brains, we mainly focused on this lipid species and verified our results with PC-PL 20 : 4, PC-PL 22 : 6 or PE-PL 22 : 6.

### 3.2. Plasmalogens Do Not Affect Gene Expression of *α*-, *β*-, and *γ*-Secretase

**Figure 2:**
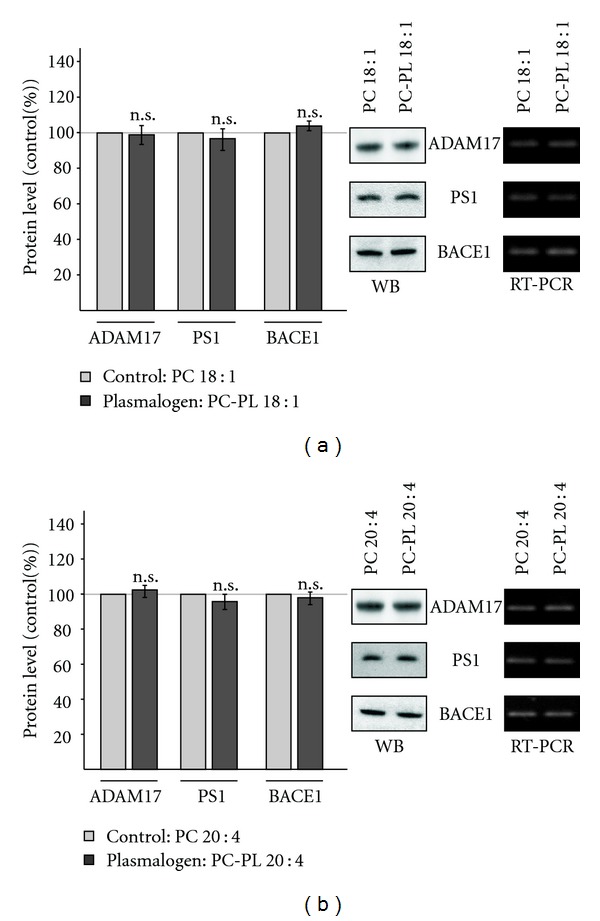
Protein level of the secretases involved in APP processing. (a) SH-SY5Y cells were incubated with PC-PL 18 : 1 and the corresponding phospholipid PC 18 : 1. Cell lysates were prepared, subjected to gel electrophoresis and Western blot (WB) analysis. Protein level of ADAM17, PS1, and BACE1 were detected with antibodies ab39162, sc-7860 and B0806, respectively. (b) Effect of PC-PL 20 : 4 on protein level of ADAM17, PS1, and BACE1 compared to the corresponding phospholipid PC 20 : 4. Detection was performed as described for (a) All quantified data represent an average of at least three independent experiments. Error bars represent standard deviation of the mean. Asterisks show the statistical significance (*P ≤ 0.05; **P ≤ 0.01 and ***P ≤ 0.001, n.s. = not significant). (a, b) Representative WBs of protein determination and representative agarose gels of RT-PCR analyis are shown.

In order to analyze whether plasmalogens affect APP cleavage by modulating gene expression of the secretases involved in the nonamyloidogenic and amyloidogenic processing of APP, we performed RT-PCR analysis of ADAM17, BACE1, and the components of the *γ*-secretase complex, PS1, PS2, Aph1a, Aph1b, nicastrin, and Pen-2 (Table 3). Therefore, cultured cells were incubated with the plasmalogens PC-PL 18 : 1 and PC-PL 20 : 4, respectively. Control cells were incubated with the corresponding phospholipid. As cellular system, we used the human neuroblastoma cell line SH-SY5Y. Significant changes in gene expression of ADAM17, BACE1, and the *γ*-secretase components were not observed, neither for PC-PL 18 : 1 nor for PC-PL 20 : 4 (Table 3). In accordance, total protein level of ADAM17, PS1 and BACE1 were not affected in presence of PC-PL 18 : 1 or PC-PL 20 : 4 (Figures 2(a) and 2(b)).

### 3.3. Influence of Plasmalogens on *β*-Secretase Activity

**Figure 3:**
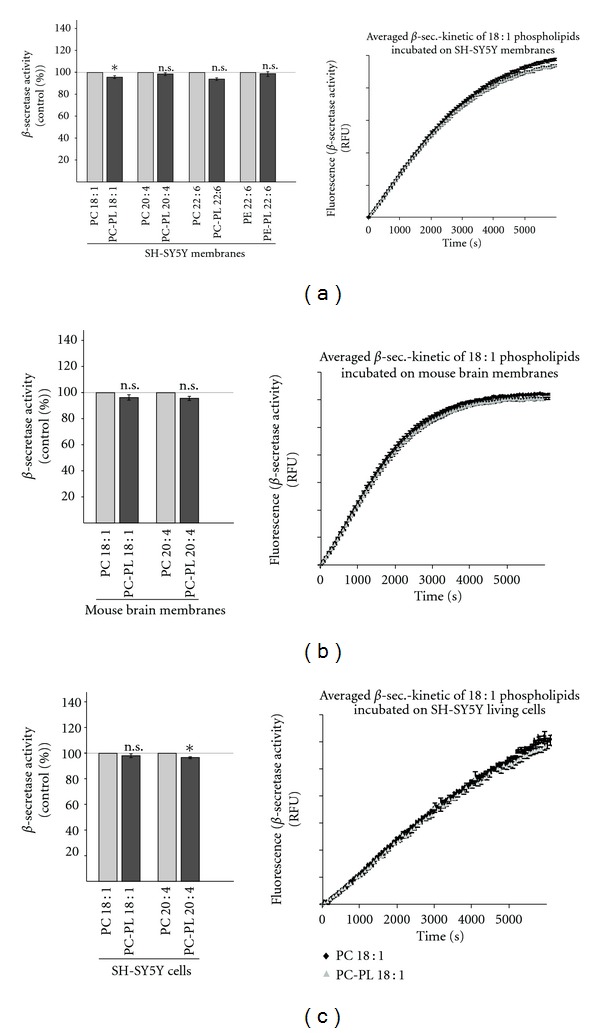
Effect of PC-PL 18 : 1, PC-PL 20 : 4, PC-PL 22 : 6 and PE-PL 22 : 6 on *β*-secretase activity. (a) Influence on *β*-secretase activity in purified membranes of human SH-SY5Y cells. Purified membranes of SH-SY5Y cells were prepared, incubated with PC-PL 18 : 1, 20 : 4, 22 : 6, or PE-PL 22 : 6 and the corresponding phospholipids (100 *μ*M), and *β*-secretase activity was determined by a fluorometric assay. A representative kinetic is shown for PC-PL 18 : 1 and PC 18 : 1. (b) Influence on *β*-secretase activity *ex vivo* in purified membranes of mouse brains. Purified membranes of mouse brains were incubated with PC-PL 18 : 1 and PC-PL 20 : 4 and the corresponding phospholipids PC 18 : 1 and PC 20 : 4 (100 *μ*M). A representative kinetic is shown for PC-PL 18 : 1 and PC 18 : 1. (c) Living SH-SY5Y cells were incubated in cell culture with PC-PL 18 : 1 and PC-PL 20 : 4 and the corresponding phospholipids in a final concentration of 100 *μ*M for 24 hours. After the incubation, purified membranes were prepared and analyzed in the *β*-secretase assay. A representative kinetic is shown for PC-PL 18 : 1 and PC 18 : 1. (a, b, c). All quantified data represent an average of at least three independent experiments. Illustration and statistical significance are as described for Figure 2.

Cleavage of APP by *β*-secretase BACE1 is the initial step in the amyloidogenic processing of APP and the generation of A*β* peptides. To examine whether plasmalogens influence *β*-secretase activity directly, we first prepared purified membranes of SH-SY5Y cells, incubated these membranes with different plasmalogens and measured *β*-secretase activity with a fluorescent *β*-secretase assay [41, 42]. PC-PL 18 : 1 and PC-PL 22 : 6 slightly directly reduced *β*-secretase activity, whereas PC-PL 20 : 4 and PE-PL 22 : 6 revealed no effect on *β*-secretase activity (Figure 3(a)). To analyze a potential direct effect of plasmalogens on *β*-secretase activity *ex vivo*, we prepared purified membranes of mouse brains for directly measuring *β*-secretase activity. PC-PL 18 : 1 as well as PC-PL 20 : 4 showed slightly, however, not significant decreased *β*-secretase activity in purified membranes of mouse brains (Figure 3(b)). To validate these results, we incubated living SH-SY5Y cells in culture with PC-PL 18 : 1 and PC-PL 20 : 4, purified the membranes of the incubated cells and determined *β*-secretase activity. The *β*-secretase activity was not significantly affected in presence of plasmalogen PC-PL 18 : 1, whereas PC-PL 20 : 4 slightly reduced *β*-secretase activity (Figure 3(c)).

### 3.4. Plasmalogens Reduce Amyloidogenic Processing by Affecting *γ*-Secretase Activity

**Figure 4:**
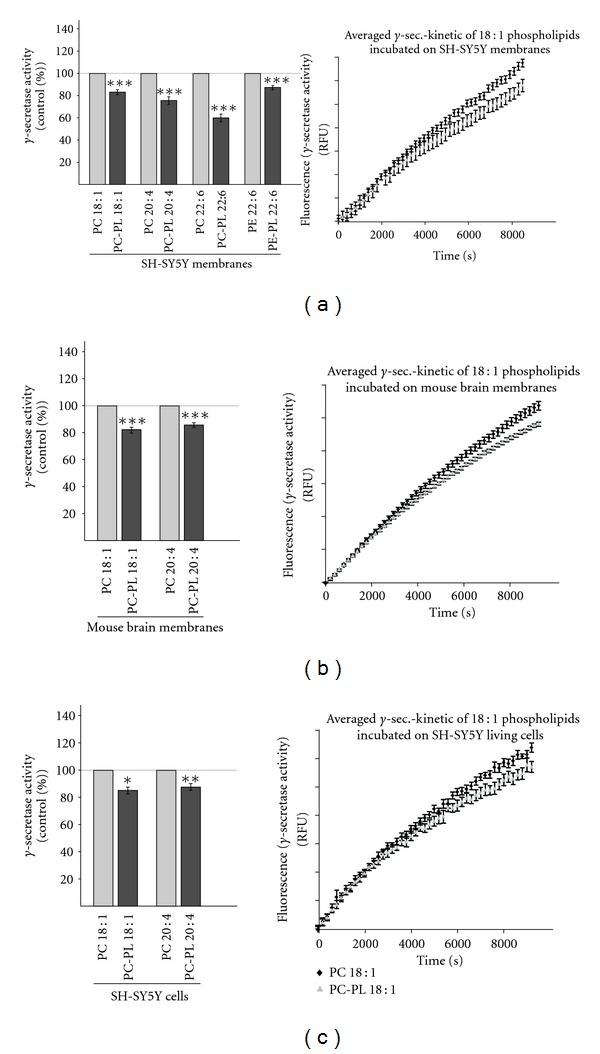
Determination of *γ*-secretase activity in the presence of PC-PL 18 : 1, PC-PL 20 : 4, PC-PL 22 : 6, and PE-PL 22 : 6. (a) Influence on *γ*-secretase activity in purified membranes of human SH-SY5Y cells. Purified membranes of SH-SY5Y cell were incubated with PC-PL 18 : 1, 20 : 4, 22 : 6, or PE-PL 22 : 6 and the corresponding phospholipids (100 *μ*M), and *γ*-secretase activity was determined by a fluorometric assay. (b) Influence on *γ*-secretase activity *ex vivo* in purified membranes of mouse brains. Purified mouse brain membranes were incubated with PC-PL 18 : 1 and PC-PL 20 : 4 and the corresponding phospholipids (100 *μ*M), and *γ*-secretase activity was determined. (c) Cultured SH-SY5Y cells were incubated with PC-PL 18 : 1 and PC-PL 20 : 4 and the corresponding phospholipids PC 18 : 1 and PC 20 : 4 for 24 hours in a final concentration of 100 *μ*M. Membranes of incubated SH-SY5Y cells were prepared and *γ*-secretase activity was determined with a fluorometric assay. (a, b, c) Representative kinetics are shown for PC-PL 18 : 1 and PC 18 : 1. All quantified data represent an average of at least three independent experiments. Illustration and statistical significance are as described for Figure 2.

As described above, we observed no or only slightly reduced *β*-secretase activity in the presence of plasmalogens, indicating that the initial step in the generation of A*β* is not or only slightly affected by plasmalogens. To evaluate a potential effect of plasmalogens on the final step in the generation of A*β* peptides, we analyzed the effect of plasmalogens on *γ*-secretase activity. Similar to the experiment for the determination of *β*-secretase activity, we first incubated purified membranes of SH-SY5Y cells with different plasmalogens, PC-PL 18 : 1, PC-PL 20 : 4, PC-PL 22 : 6, and PE-PL 22 : 6, and directly measured *γ*-secretase activity. All analyzed plasmalogens PC-PL 18 : 1, PC-PL 20 : 4, PC-PL 22 : 6, and PE-PL 22 : 6 significantly reduced *γ*-secretase activity (Figure 4(a)). The strongest effect was observed for PC-PL 22 : 6, which reduced *γ*-secretase activity to 60%. In agreement with these findings, PC-PL 18 : 1 and PC-PL 20 : 4 also significantly reduced *γ*-secretase activity of *ex vivo* purified membranes from mouse brains to 80% (Figure 4(b)). Similar results were obtained when SH-SY5Y cells were cultured in presence of PC-PL 18 : 1 or PC-PL 20 : 4 (Figure 4(c)).

### 3.5. Influence of Plasmalogens on *α*-Secretase Activity

**Figure 5:**
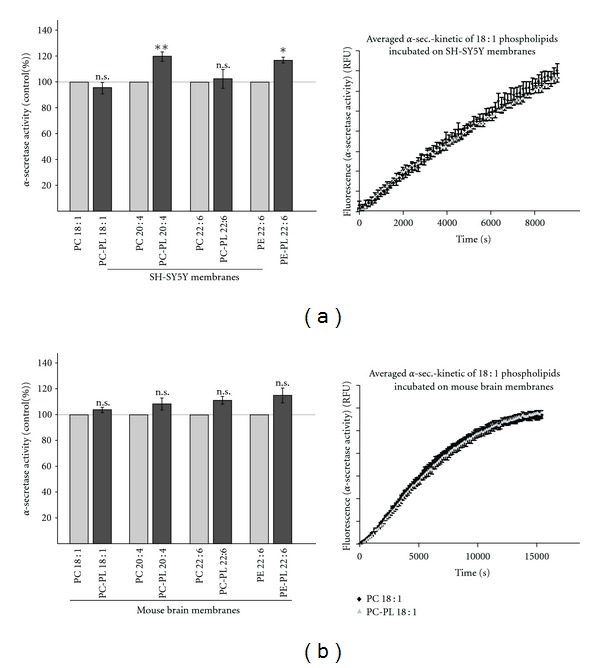
Effect of PC-PL 18 : 1, PC-PL 20 : 4, PC-PL 22 : 6, and PE-PL 22 : 6 on *α*-secretase activity. PC-PL 18 : 1, PC-PL 20 : 4, PC-PL 22 : 6 and PE-PL 22 : 6 and the corresponding phospholipids (100 *μ*M) were incubated on purified membranes of (a) human SH-SY5Y cells and (b) mouse brains, and *α*-secretase activity was determined as described in materials and methods. (a, b) Representative kinetics were shown for PC-PL 18 : 1 and PC 18 : 1. All quantified data represent an average of at least three independent experiments. Illustration and statistical significance as described for Figure 2.

In contrast to *β*-secretase cleavage of APP which generates the N-terminus of A*β*, APP shedding by *α*-secretase prevents the formation of toxic A*β* peptides, because *α*-secretase cleaves APP within the A*β* domain [32, 33, 43]. In order to evaluate whether plasmalogens also affect nonamyloidogenic processing of APP, we directly measured *α*-secretase activity in purified membranes of SH-SY5Y cells and mouse brains. Plasmalogens PC-PL 18 : 1 and PC-PL 22 : 6 showed no effect on *α*-secretase activity, whereas PC-PL 20 : 4 and PE-PL 22 : 6 significantly increased *α*-secretase activity by 10% to 20% (Figure 5(a)) in purified membranes of SH-SY5Y cells. However, *α*-secretase activity was not significantly elevated for any of the analyzed plasmalogens, PC-PL 18 : 1, PC-PL 20 : 4, PC-PL 22 : 6, and PE-PL 22 : 6 when we used purified membranes of mouse brains instead of SH-SY5Y membranes.

### 3.6. Influence of PC-PL on *γ*-Secretase Activity in Human AD Brains

**Figure 6:**
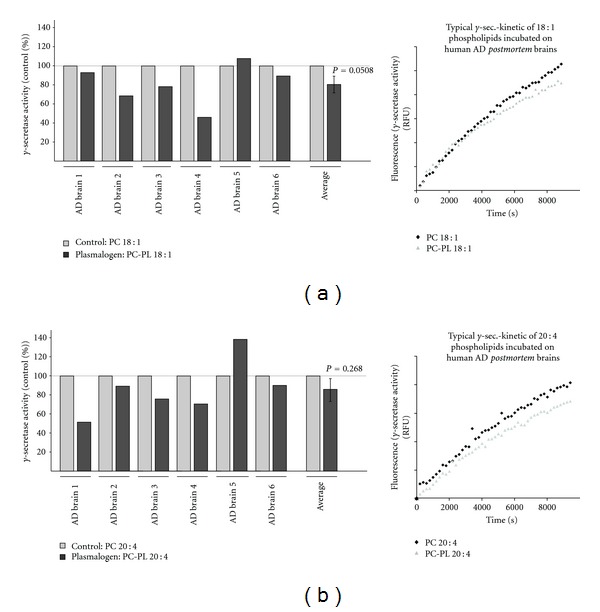
Effect of PC-PL 18 : 1 and PC-PL 20 : 4 on *γ*-secretase activity in purified membranes of human AD *postmortem* brains. Membranes of six human AD *postmortem* brain samples were purified and incubated with (a) PC-PL 18 : 1 and the corresponding phospholipid PC 18 : 1 or (b) PC-PL 20 : 4 and PC 20 : 4 as control. PC-PLs and corresponding phospholipids were incubated in a final concentration of 100 *μ*M and *γ*-secretase activity was determined with a fluorometric assay. (a, b) Representative kinetics were shown. Illustration and statistical significance are as described for Figure 2.

Above findings indicate that PC-PL reduce amyloidogenic processing of APP by affecting *γ*-secretase activity. The significantly reduced levels of PC-PL in the analyzed AD *postmortem* brains might therefore in return increase *γ*-secretase activity, leading to the massive generation of A*β* peptides, one of the characteristic hallmarks for AD. To reveal whether an increase in plasmalogens is in principle able to decrease *γ*-secretase in human AD brains we prepared purified membranes of six AD *postmortem* brains and incubated these membranes with PC-PL 18 : 1 and PC-PL 20 : 4 and the corresponding PC phospholipids. As described above, we found that PC-PL 18 : 1 is one of the species which is mostly changed in AD brains and was therefore selected for the incubation experiments. Indeed supplementation with PC-PL 18 : 1 reduced *γ*-secretase activity in five out of six AD brains (Figures 6(a) and 6(b)). A similar result was obtained by incubation with PC-PL 20 : 4. Interestingly, the same AD brain, which showed instead of decreased, increased *γ*-secretase activity for PC-PL 18 : 1 also showed increased *γ*-secretase activity for PC-PL 20 : 4 as well (AD brain 5, Figure 6). However, in total, PC-PL 18 : 1 reduced *γ*-secretase activity to 80%, statistical analysis revealed a *P* value of 0.0508 (Figure 6(a)). For PC-PL 20 : 4, the mean reduction in *γ*-secretase activity was in a similar range with a *P* value of 0.268, when the results of all six AD brains were combined (Figure 6(b)).

## 4. Discussion

Plasmalogens are a subclass of glycerophospholipids characterized by the presence of an enol ether substituent at the sn-1 position of the glycerol backbone [1]. Beside phosphoethanolamine-plasmalogens (PE-PL), reported to be the major plasmalogens in the brain, a further plasmalogen species, phosphatidylcholine-plasmalogens (PC-PL) occur in brain [3, 44]. Plasmalogens are common constituents of cellular membranes, most abundant in brain and heart, with important functions like signal transduction, ion transport, membrane fusion, cell-cell communication, and cholesterol dynamics (reviewed in: [3]). Alterations in the lipid composition of cellular membranes affect membrane fluidity and a number of cellular functions [45] and occur in several diseases, including AD [15, 17, 46–48], Parkinson's Disease [49], Creutzfeldt-Jakob disease [50, 51], Gaucher disease [52], and Fabry disease [53, 54]. Recent studies have shown that several lipids influence the proteolytic processing of APP. Cholesterol and GM1 are reported to increase the generation of A*β* [55–57], whereas docosahexaenoic acid and sphingomyelin decrease amyloidogenic processing of APP [42, 58]. Beside their influence on the generation of A*β*, altered lipid composition might also affect the recently identified physiological function of APP. Beside the neuroprotective and memory enhancing effect of *α*-secreted APP [59–61], we and others could show that A*β* and the intracellular domain of APP (AICD), which is beside A*β* released by *γ*-secretase cleavage of *β*-CTF, regulate lipid homeostasis and gene transcription [39, 58, 62, 63]. AICD has been reported to affect gene transcription of several proteins, including APP, BACE, GSK3*β* [62], serine-palmitoyl-CoA transferase [63], and alkyl-dihydroxyacetonephosphate-synthase (AGPS) [39], a rate limiting enzyme in plasmalogen synthesis. Although plasmalogen levels are altered in AD brain samples and are major constituents of neuronal membranes, so far, it is not known whether plasmalogens affect the proteolytic processing of APP. Our studies on the proteolytic processing of APP revealed that plasmalogens decrease the amyloidogenic processing of APP. The detailed analysis of the secretases involved in A*β* generation, *β*- and *γ*-secretase, revealed that plasmalogens decrease amyloidogenic processing by reducing *γ*-secretase activity, whereas no or only a very slight reduction in the enzymatic activity of *β*-secretase was determined. All PC-PL and PE-PL species analyzed showed a highly significant decrease in *γ*-secretase activity to 60%–85% compared to the corresponding phospholipid lacking the enol ether. All plasmalogens independent of the fatty acid showed a decrease in *γ*-secretase activity further emphasizing that the observed effect is due to the enol ether and not to the fatty acid. Analyzed protein levels of PS1 and BACE1 in presence of PC-PL 18 : 1 and PC-PL 20 : 4 and RT-PCR analysis of PC-PL 18 : 1 or PC-PL 20 : 4 incubated cells showed that plasmalogens do not affect gene expression of the secretases involved in amyloidogenic processing of APP. This result is in line with our finding that PC-PL 18 : 1 and PC-PL 20 : 4 incubated cultured SH-SY5Y cells showed a similar reduction in *γ*-secretase activity like purified membranes of SH-SY5Y cells incubated with PC-PL 18 : 1 or PC-PL 20 : 4, further strengthening our finding that plasmalogens directly reduce the enzymatic activity of *γ*-secretase and that it is unlikely that indirect effects of plasmalogens might influence *γ*-secretase activity. The *α*-secretase activity, which prevents the formation of A*β* peptides, was in contrast to *γ*-secretase diversely affected by plasmalogens. Whereas PC-PL 18 : 1 and PC-PL 22 : 6 showed no significant differences in *α*-secretase activity compared to PC 18 : 1 and PC 22 : 6, respectively, PC-PL 20 : 4 and PE-PL 22 : 6 significantly increased directly *α*-secretase activity compared to the corresponding phospholipids suggesting that the fatty acids or the phospholipid headgroups are at least able to modulate the effect of the enol ether on *α*-secretase activity. However, this increase in *α*-secretase activity was only obtained when purified membranes of SH-SY5Y cells were used for the *α*-secretase assay, whereas on purified membranes of mouse brains all plasmalogens showed no significant changes in *α*-secretase activity. Many matrix metalloproteases (MMPs) are known to contribute to the *α*-secretase activity initiating the nonamyloidogenic pathway [32–34, 64]. These MMPs are differentially expressed in different cell lines or tissues [65–67]. The different effect for PC-PL 20 : 4 and PE-PL 22 : 6 might therefore be a result of different *α*-secretase composition in SH-SY5Y cells and mouse brains. Similar to the effect on the protein level and RNA level of BACE1 and the *γ*-secretase components, PC-PL 18 : 1 and PC-PL 20 : 4 revealed no changes in gene transcription of ADAM17. Because of the cell-type-specific effect of some plasmalogen species, we cannot exclude that plasmalogens influence some other MMPs, which contribute to *α*-secretase activity. However these effects cannot be observed for all tested plasmalogens pointing out that in respect to *α*-secretase the enol ether has only a minor or modulating effect.

To test if our findings are relevant in AD, we analyzed whether AD *postmortem* brains show altered levels of PC-PL and PE-PL. For PE-PL several studies have revealed that PE-PL level are reduced in AD brain [15, 17, 23, 68]. However, one study reported no differences or even a slight increase in PE-PL level in AD [25]. By analyzing 37 AD *postmortem* brain samples compared to 21 control brains not affected by AD, we found PE-PL level to be reduced in AD brains; however, statistical analysis of the single PE-PL species revealed no significance. These findings are in line with the reduced PE-PL level reported by Ginsberg et al. [15] and Han et al. [24, 68] and our recent study that revealed also reduced PC-PL level in AD *postmortem* brain [40]. Interestingly, PC-PL level were significantly decreased in AD *postmortem* brain, indicating that PC-PL might play an important role in the development of AD, although PC-PL are less abundant in neuronal membranes compared to PE-PL [44]. The importance of PC-PL is further substantiated by the results obtained for *γ*-secretase activities. As PC-PL level showed the most prominent reduction in AD brains and AD brains show increased A*β* generation and accumulation, we tested whether incubation of PC-PL 18 : 1 and PC-PL 20 : 4 on purified membranes of human AD *postmortem* brains might also reduce *γ*-secretase activity. Indeed, supplementation with PC-PL 18 : 1 or PC-PL 20 : 4 on AD brain samples reduced *γ*-secretase activity in five out of six analyzed AD brains, further strengthening the importance of PC-PL in APP processing and probably the development of AD. Incubating plasmalogens *ex vivo* on human *postmortem* material has its clear limitations; further studies are required to clarify whether an increase in plasmalogen levels are a suitable target, which might have a positive impact in AD.

However, the importance of plasmalogens on the pathogenesis of AD is further substantiated by our recent finding that AGPS, a rate-limiting enzyme in plasmalogen synthesis, is regulated by APP processing [39]. Increased A*β* levels as observed in AD lead to peroxisomal dysfunction and reduced AGPS protein stability, resulting in reduced AGPS protein level and reduced plasmalogen *de novo* synthesis [39]. Furthermore, A*β* peptides have been shown to increase the formation of reactive oxidative species [69–74], also reducing plasmalogen levels, because plasmalogens are susceptible to oxidative stress and function as antioxidants. Reduced plasmalogen levels in AD might also be a result of increased phospholipase A2 activity. Sanchez-Mejia et al. recently reported that A*β* stimulates phospholipase A2 [75, 76], responsible for the degradation of plasmalogens. Increased levels of A*β* peptides therefore, decrease plasmalogen levels by reducing AGPS protein stability, increasing oxidative stress and activation of phospholipase A2. Therefore, in AD, a vicious cycle between APP processing and plasmalogen level occurs. A*β* peptides reduce the plasmalogen level and reduced plasmalogen level directly increase *γ*-secretase activity leading to an even stronger production of A*β* peptides. In summary, our findings indicate that plasmalogens might play a crucial role in the development of AD and that a delicate balance in lipid composition of cellular membranes is important for neuronal function.

## Supplementary Material

The supplemental information contains the cytotoxicity measurements after PC-PL and PE-PL incubation. Further, specificity controls of secretase assays for *α*-, *β*- and *γ*-secretase were presented. All methods used in the supplemental information are described in the Material and Method section within the manuscript.

## Figures and Tables

**Table 1:** List of all human brains (*n* = 58) used for analysis. Human brain samples were kindly provided from BrainNet (Munich). In total, we used 58 human brain samples from 21 control and 37 AD patients. Brains were obtained from patients with an age at death between 61 and 88 years, and no significant differences in age and gender were observed between control (mean 75 years) and AD patients (mean 78 years) group. Abbreviations used are AD = Alzheimer's disease; F = female; M = male; CERAD = the consortium to establish a registry for AD, standardizing procedures for the evaluation and diagnosis if patients with AD. A, B, C, 0 as described in http://cerad.mc.duke.edu/; Braak and Braak = Braak and Braak stage of AD; H. Braak and E. Braak stages [36]; FR = frontal cortex; n.d. = not determined.

#	Age at death	Sex	Diagnosis	*Postmortem* delay [h]	Braak and Braak	CERAD	Brain region
Con #01	69	n.d.	Control	14	n.d.	n.d.	n.d.
Con #02	77	F	Control	n.d.	II	A	FR
Con #03	61	M	Control	24	0	0	FR
Con #04	85	F	Control	20	I	0	FR
Con #05	80	F	Control	n.d.	III-IV	0	FR
Con #06	75	M	Control	27	II	0	FR
Con #07	71	M	Control	23	0-I	0	FR
Con #08	79	n.d.	Control	20	n.d.	n.d.	n.d.
Con #09	62	n.d.	Control	48	n.d.	n.d.	n.d.
Con #10	88	F	Control	48	I-II	B	FR
Con #11	64	n.d.	Control	15	n.d.	n.d.	n.d.
Con #12	69	n.d.	Control	30	n.d.	n.d.	n.d.
Con #13	83	F	Control	22	II	0	FR
Con #14	74	n.d.	Control	23	n.d.	n.d.	n.d.
Con #15	85	M	Control	25	III-IV	B	FR
Con #16	76	F	Control	26	III-IV	C	FR
Con #17	87	M	Control	48	I-II	0	FR
Con #18	71	n.d.	Control	48	n.d.	n.d.	n.d.
Con #19	75	F	Control	24	III-IV	B	FR
Con #20	77	F	Control	20	II	C	FR
Con #21	63	M	Control	18	I	0	FR

AD #01	83	M	AD	22	VI	C	FR
AD #02	78	F	AD	21	VI	C	FR
AD #03	76	M	AD	14	V	B	FR
AD #04	88	F	AD	39	VI	C	FR
AD #05	67	F	AD	49	V-VI	C	FR
AD #06	82	F	AD	33	V	C	FR
AD #07	80	M	AD	12	V	C	FR
AD #08	75	M	AD	24	VI	C	FR
AD #09	74	M	AD	50	V-VI	C	FR
AD #10	83	M	AD	37,5	VI	C	FR
AD #11	80	M	AD	13	V	C	FR
AD #12	88	F	AD	36	V	C	FR
AD #13	73	M	AD	24	V-VI	C	FR
AD #14	62	M	AD	n.d.	VI	C	FR
AD #15	70	M	AD	39	VI	C	FR
AD #16	81	n.d.	AD	n.d.	n.d.	n.d.	FR
AD #17	75	F	AD	12	VI	C	FR
AD #18	73	n.d.	AD	n.d.	n.d.	n.d.	FR
AD #19	78	F	AD	n.d.	V-VI	C	FR
AD #20	79	n.d.	AD	18	>V	C	FR
AD #21	86	n.d.	AD	42	>V	C	n.d.
AD #22	85	F	AD	n.d.	IV	C	FR
AD #23	75	n.d.	AD	18	VI	C	n.d.
AD #24	80	F	AD	48	V	C	FR
AD #25	73	F	AD	n.d.	V-VI	C	FR
AD #26	85	F	AD	n.d.	III	C	FR
AD #27	80	n.d.	AD	5	>V	C	n.d.
AD #28	78	F	AD	n.d.	VI	C	FR
AD #29	87	M	AD	4	V	C	FR
AD #30	65	F	AD	48	V-VI	C	FR
AD #31	82	F	AD	14	V-VI	C	FR
AD #32	76	F	AD	24	V-VI	C	FR
AD #33	79	F	AD	20	V	C	n.d.
AD #34	87	M	AD	26	V	C	FR
AD #35	68	F	AD	n.d.	VI	C	FR
AD #36	85	n.d.	AD	n.d.	n.d.	n.d.	FR
AD #37	83	M	AD	48	V	C	FR

**Table 2:** Analyzed PC-PL and PE-PL species in human AD *postmortem* brains compared to healthy individuals. Levels of all analyzed PC-PL and PE-PL species were reduced in human AD *postmortem* brains. Statistical significance was determined by two-tailed Students *t*-test. SEM = standard deviation of the mean.

Metabolite	% (compared to control)	SEM	*t*-test
PC-PL 16 : 0/18 : 1 (PC-PL 34 : 1)	64,55	1,654	0,0011
PC-PL 18 : 0/18 : 1 (PC-PL 36 : 1)	68,93	1,814	0,0005
PC-PL 18 : 1/18 : 1 (PC-PL 36 : 2)	49,17	1,452	0,0007
PC-PL 16 : 0/20 : 4 (PC-PL 36 : 4)	81,56	2,681	0,0483
PC-PL 18 : 0/20 : 4 (PC-PL 38 : 4)	85,02	1,930	0,0051
PC-PL 18 : 1/20 : 4 (PC-PL 38 : 5)	84,58	3,744	0,1105
PC-PL 16 : 0/22 : 6 (PC-PL 38 : 6)	88,21	2,925	0,0735
PC-PL 18 : 0/22 : 6 (PC-PL 40 : 6)	88,99	2,388	0,0184

PE-PL 18 : 1/18 : 1 (PE-PL 36 : 2)	83,18	7,252	0,2152
PE-PL 16 : 0/20 : 4 (PE-PL 36 : 4)	78,67	7,181	0,1631
PE-PL 18 : 0/20 : 4 (PE-PL 38 : 4)	80,55	6,795	0,1540
PE-PL 18 : 1/20 : 4 (PE-PL 38 : 5)	93,96	6,194	0,5656
PE-PL 16 : 0/22 : 6 (PE-PL 38 : 6)	94,07	6,250	0,5752
PE-PL 18 : 0/22 : 6 (PE-PL 40 : 6)	86,24	5,190	0,2049

**Table 3:** RT-PCR analysis of *α*-secretase ADAM17, the *γ*-secretase components and *β*-secretase BACE1 in presence of PC-PL 18 : 1 and PC-PL 20 : 4 compared to the corresponding phospholipids PC 18 : 1 and PC 20 : 4 as control. The abbreviations set are ADAM17 = *α*-secretase, PSEN1 = presenilin1, PSEN2 = presenilin2, APH1a = anterior pharynx-defective 1a, APH1b = anterior pharynx-defective 1b, NCSTN = nicastrin, PSENEN2 = presenilin enhancer 2, and SEM = standard deviation of the mean. Statistical significance was determined by two-tailed Student's *t*-test.

		Gene	% (compared to control)	SEM	*t*-test
PC-PL 18 : 0/18 : 1	*α*-secretase	ADAM17	110,65	17,775	0,5658
	*γ*-secretase	PSEN1	96,32	11,721	0,7574
		PSEN2	103,59	13,585	0,7981
		APH1a	87,01	23,043	0,5884
		APH1b	106,43	18,578	0,7380
		NCSTN	87,42	20,414	0,5548
		PSENEN2	108,75	14,912	0,5734
	*β*-secretase	BACE1	104,24	22,534	0,8554

PC-PL 18 : 0/20 : 4	*α*-secretase	ADAM17	90,44	4,919	0,0878
	*γ*-secretase	PSEN1	94,97	9,865	0,6162
		PSEN2	110,28	10,516	0,3568
		APH1a	86,45	8,927	0,1676
		APH1b	92,58	12,544	0,5707
		NCSTN	85,93	8,304	0,1287
		PSENEN2	98,99	7,433	0,8949
	*β*-secretase	BACE1	92,90	9,413	0,4607

## References

[1] Horrocks LA, Sharma M, Hawthorne JN, Answell GB (1982). Plasmalogen and O-alkyl glycerophospholipids. *Phospholipids*.

[2] Gross RW (1985). Identification of plasmalogen as the major phospholipid constituent of cardiac sarcoplasmic reticulum. *Biochemistry*.

[3] Farooqui AA, Horrocks LA (2001). Plasmalogens: workhorse lipids of membranes in normal and injured neurons and glia. *Neuroscientist*.

[4] Zoeller RA, Morand OH, Raetz CR (1988). A possible role for plasmalogens in protecting animal cells against photosensitized killing. *Journal of Biological Chemistry*.

[5] Breckenridge WC, Morgan IG, Zanetta JP, Vincendon G (1973). Adult rat brain synaptic vesicles. II. Lipid composition. *Biochimica et Biophysica Acta*.

[6] Glaser PE, Gross RW (1994). Plasmenylethanolamine facilitates rapid membrane fusion: a stopped-flow kinetic investigation correlating the propensity of a major plasma membrane constituent to adopt an HII phase with its ability to promote membrane fusion. *Biochemistry*.

[7] Ford DA, Hale CC (1996). Plasmalogen and anionic phospholipid dependence of the cardiac sarcolemmal sodium-calcium exchanger. *FEBS Letters*.

[8] Duhm J, Engelmann B, Schonthier UM, Streich S (1993). Accelerated maximal velocity of the red blood cell Na+/K+ pump in hyperlipidemia is related to increase in 1-palmitoyl, 2-arachidonoyl-plasmalogen phosphatidylethanolamine. *Biochimica et Biophysica Acta*.

[9] Young C, Gean PW, Chiou LC, Shen YZ (2000). Docosahexaenoic acid inhibits synaptic transmission and epileptiform activity in the rat hippocampus. *Synapse*.

[10] Nagan N, Hajra AK, Larkins LK (1998). Isolation of a Chinese hamster fibroblast variant defective in dihydroxyacetonephosphate acyltransferase activity and plasmalogen biosynthesis: use of a novel two-step selection protocol. *Biochemical Journal*.

[11] Mandel H, Sharf R, Berant M, Wanders RJ, Vreken P, Aviram M (1998). Plasmalogen phospholipids are involved in HDL-mediated cholesterol efflux: insights from investigations with plasmalogen-deficient cells. *Biochemical and Biophysical Research Communications*.

[12] Farooqui AA, Horrocks LA (2001). Plasmalogens, phospholipase A2, and docosahexaenoic acid turnover in brain tissue. *Journal of Molecular Neuroscience*.

[13] Farooqui AA, Rosenberger TA, Horrocks LA, Yehuda S, Mostofsky DI (1997). Arachidonic acid, neurotrauma, and neurodegenerative diseases. *Handbook of Essential Fatty Acid Biology*.

[14] Yang HC, Farooqui AA, Horrocks LA (1996). Plasmalogen-selective phospholipase A2 and its role in signal transduction. *Journal of Lipid Mediators and Cell Signalling*.

[15] Ginsberg L, Rafique S, Xuereb JH, Rapoport SI, Gershfeld NL (1995). Disease and anatomic specificity of ethanolamine plasmalogen deficiency in Alzheimer’s disease brain. *Brain Research*.

[16] Wells K, Farooqui AA, Liss L, Horrocks LA (1995). Neural membrane phospholipids in Alzheimer disease. *Neurochemical Research*.

[17] Guan Z, Wang Y, Cairns NJ, Lantos PL, Dallner G, Sindelar PJ (1999). Decrease and structural modifications of phosphatidylethanolamine plasmalogen in the brain with Alzheimer disease. *Journal of Neuropathology and Experimental Neurology*.

[18] Demediuk P, Saunders RD, Anderson DK (1985). Membrane lipid changes in laminectomized and traumatized cat spinal cord. *Proceedings of the National Academy of Sciences of the United States of America*.

[19] Viani P, Zini I, Cervato G, Biagini G, Agnati LF, Cestaro B (1995). Effect of endothelin-1 induced ischemia on peroxidative damage and membrane properties in rat striatum synaptosomes. *Neurochemical Research*.

[20] Zhang JP, Sun GY (1995). Free fatty acids, neutral glycerides, and phosphoglycerides in transient focal cerebral ischemia. *Journal of Neurochemistry*.

[21] Schedin S, Sindelar PJ, Pentchev P, Brunk U, Dallner G (1997). Peroxisomal impairment in Niemann-Pick type C disease. *Journal of Biological Chemistry*.

[22] Yanagihara T, Cumings JN (1969). Alterations of phospholipids, particularly plasmalogens, in the demyelination of multiple sclerosis as compared with that of cerebral oedema. *Brain*.

[23] Farooqui AA, Rapoport SI, Horrocks LA (1997). Membrane phospholipid alterations in Alzheimer’s disease: deficiency of ethanolamine plasmalogens. *Neurochemical Research*.

[24] Han X, Holtzman DM, McKeel DW (2001). Plasmalogen deficiency in early Alzheimer’s disease subjects and in animal models: molecular characterization using electrospray ionization mass spectrometry. *Journal of Neurochemistry*.

[25] Pettegrew JW, Panchalingam K, Hamilton RL, Mcclure RJ (2001). Brain membrane phospholipid alterations in Alzheimer’s disease. *Neurochemical Research*.

[26] Masters CL, Simms G, Weinman NA (1985). Amyloid plaque core protein in Alzheimer disease and Down syndrome. *Proceedings of the National Academy of Sciences of the United States of America*.

[27] Selkoe DJ (2004). Cell biology of protein misfolding: the examples of Alzheimer’s and Parkinson’s diseases. *Nature Cell Biology*.

[28] Haass C (2004). Take five—BACE and the *γ*-secretase quartet conduct Alzheimer’s amyloid *β*-peptide generation. *EMBO Journal*.

[29] Sinha S, Anderson JP, Barbour R (1999). Purification and cloning of amyloid precursor protein *β*-secretase from human brain. *Nature*.

[30] Steiner H, Fluhrer R, Haass C (2008). Intramembrane proteolysis by *γ*-secretase. *Journal of Biological Chemistry*.

[31] Wakabayashi T, De Strooper B (2008). Presenilins: members of the *γ*-secretase quartets, but part-time soloists too. *Physiology*.

[32] Buxbaum JD, Liu KN, Luo Y (1998). Evidence that tumor necrosis factor *α* converting enzyme is involved in regulated *α*-secretase cleavage of the Alzheimer amyloid protein precursor. *Journal of Biological Chemistry*.

[33] Lammich S, Kojro E, Postina R (1999). Constitutive and regulated *α*-secretase cleavage of Alzheimer’s amyloid precursor protein by a disintegrin metalloprotease. *Proceedings of the National Academy of Sciences of the United States of America*.

[34] Koike H, Tomioka S, Sorimachi H (1999). Membrane-anchored metalloprotease MDC9 has an *α*-secretase activity responsible for processing the amyloid precursor protein. *Biochemical Journal*.

[35] Allinson TM, Parkin ET, Turner AJ, Hooper NM (2003). ADAMs family members as amyloid precursor protein *α*-secretases. *Journal of Neuroscience Research*.

[36] Braak H, Braak E (1991). Neuropathological stageing of Alzheimer-related changes. *Acta Neuropathologica*.

[37] Smith PK, Krohn RI, Hermanson GT (1985). Measurement of protein using bicinchoninic acid. *Analytical Biochemistry*.

[38] Livak KJ, Schmittgen TD (2001). Analysis of relative gene expression data using real-time quantitative PCR and the 2(-Delta Delta C(T)) Method. *Methods*.

[39] Grimm MO, Kuchenbecker J, Rothhaar TL (2011). Plasmalogen synthesis is regulated via alkyl-dihydroxyacetonephosphate-synthase by amyloid precursor protein processing and is affected in Alzheimer's disease. *Journal of Neurochemistry*.

[40] Grimm MO, Grösgen S, Riemenschneider M, Tanila H, Grimm HS, Hartmann T (2011). From brain to food: analysis of phosphatidylcholins, lyso-phosphatidylcholins and phosphatidylcholin-plasmalogens derivates in Alzheimer's disease human post mortem brains and mice model via mass spectrometry. *Journal of Chromatography A*.

[41] Grimm MO, Grimm HS, Tomic I, Beyreuther K, Hartmann T, Bergmann C (2008). Independent inhibition of Alzheimer disease *β*- and *γ*-secretase cleavage by lowered cholesterol levels. *Journal of Biological Chemistry*.

[42] Grimm MO, Kuchenbecker J, Grösgen S (2011). Docosahexaenoic acid reduces amyloid *β* production via multiple pleiotropic mechanisms. *Journal of Biological Chemistry*.

[43] Allinson TM, Parkin ET, Condon TP (2004). The role of ADAM10 and ADAM17 in the ectodomain shedding of angiotensin converting enzyme and the amyloid precursor protein. *European Journal of Biochemistry*.

[44] Miyazawa T, Kanno S, Eitsuka T, Nakagawa K, Yanagita Y, Knapp, HR, Huang YS (2006). Plasmalogen: a short review ans newly-discovered functions. *Dietary Fats and Risk of Chronic Disease*.

[45] Spector AA, Yorek MA (1985). Membrane lipid composition and cellular function. *Journal of Lipid Research*.

[46] Prasad MR, Lovell MA, Yatin M, Dhillon H, Markesbery WR (1998). Regional membrane phospholipid alterations in Alzheimer’s disease. *Neurochemical Research*.

[47] Goodenowe DB, Cook LL, Liu J (2007). Peripheral ethanolamine plasmalogen deficiency: a logical causative factor in Alzheimer’s disease and dementia. *Journal of Lipid Research*.

[48] Wood PL, Mankidy R, Ritchie S (2010). Circulating plasmalogen levels and Alzheimer disease assessment scale-cognitive scores in Alzheimer patients. *Journal of Psychiatry and Neuroscience*.

[49] Fabelo N, Martin V, Santpere G (2011). Severe alterations in lipid composition of frontal cortex lipid rafts from Parkinson's disease and incidental Parkinson's disease. *Molecular Medicine*.

[50] Tamai Y, Kojima H, Ikuta F, Kumanishi T (1978). Alterations in the composition of brain lipids in patients with Creutzfeldt-Jakob disease. *Journal of the Neurological Sciences*.

[51] Federico A, Annunziata P, Malentacchi G (1980). Neurochemical changes in Creutzfeldt-Jakob disease. *Journal of Neurology*.

[52] Hein LK, Duplock S, Hopwood JJ, Fuller M (2008). Lipid composition of microdomains is altered in a cell model of Gaucher disease. *Journal of Lipid Research*.

[53] Hozumi I, Nishizawa M, Ariga T, Miyatake T (1990). Biochemical and clinical analysis of accumulated glycolipids in symptomatic heterozygotes of angiokeratoma corporis diffusum (Fabry’s disease) in comparison with hemizygotes. *Journal of Lipid Research*.

[54] Maalouf K, Jia J, Rizk S (2010). A modified lipid composition in Fabry disease leads to an intracellular block of the detergent-resistant membrane-associated dipeptidyl peptidase IV. *Journal of Inherited Metabolic Disease*.

[55] Fassbender K, Simons M, Bergmann C (2001). Simvastatin strongly reduces levels of Alzheimer’s disease *β*-amyloid peptides A*β*42 and A*β*40 in vitro and in vivo. *Proceedings of the National Academy of Sciences of the United States of America*.

[56] Zha Q, Ruan Y, Hartmann T, Beyreuther K, Zhang D (2004). GM1 ganglioside regulates the proteolysis of amyloid precursor protein. *Molecular Psychiatry*.

[57] Wolozin B (2004). Cholesterol and the biology of Alzheimer’s disease. *Neuron*.

[58] Grimm MO, Grimm HS, Patzold AJ (2005). Regulation of cholesterol and sphingomyelin metabolism by amyloid-*β* and presenilin. *Nature Cell Biology*.

[59] Furukawa K, Sopher BL, Rydel RE (1996). Increased activity-regulating and neuroprotective efficacy of *α*-secretase-derived secreted amyloid precursor protein conferred by a C-terminal heparin-binding domain. *Journal of Neurochemistry*.

[60] Meziane H, Dodart JC, Mathis C (1998). Memory-enhancing effects of secreted forms of the *β*-amyloid precursor protein in normal and amnestic mice. *Proceedings of the National Academy of Sciences of the United States of America*.

[61] Mattson MP, Guo ZH, Geiger JD (1999). Secreted form of amyloid precursor protein enhances basal glucose and glutamate transport and protects against oxidative impairment of glucose and glutamate transport in synaptosomes by a cyclic GMP-mediated mechanism. *Journal of Neurochemistry*.

[62] von Rotz RC, Kohli BM, Bosset J (2004). The APP intracellular domain forms nuclear multiprotein complexes and regulates the transcription of its own precursor. *Journal of Cell Science*.

[63] Grimm MO, Grösgen S, Rothhaar TL (2011). Intracellular APP domain regulates serine-palmitoyl-CoA transferase expression and is affected in alzheimer's disease. *International Journal of Alzheimer's Disease*.

[64] Blacker M, Noe MC, Carty TJ, Goodyer CG, LeBlanc AC (2002). Effect of tumor necrosis factor-*α* converting enzyme (TACE) and metalloprotease inhibitor on amyloid precursor protein metabolism in human neurons. *Journal of Neurochemistry*.

[65] Karkkainen I, Rybnikova E, Pelto-Huikko M, Huovila AP (2000). Metalloprotease-disintegrin (ADAM) genes are widely and differentially expressed in the adult CNS. *Molecular and Cellular Neurosciences*.

[66] Llano E, Adam G, Pendas AM (2002). Structural and enzymatic characterization of Drosophila Dm2-MMP, a membrane-bound matrix metalloproteinase with tissue-specific expression. *Journal of Biological Chemistry*.

[67] Lin J, Yan X, Markus A, Redies C, Rolfs A, Luo J (2010). Expression of seven members of the ADAM family in developing chicken spinal cord. *Developmental Dynamics*.

[68] Han X (2010). Multi-dimensional mass spectrometry-based shotgun lipidomics and the altered lipids at the mild cognitive impairment stage of Alzheimer’s disease. *Biochimica et Biophysica Acta*.

[69] Subbarao KV, Richardson JS, Ang LS (1990). Autopsy samples of Alzheimer’s cortex show increased peroxidation in vitro. *Journal of Neurochemistry*.

[70] Mark RJ, Pang Z, Geddes JW, Uchida K, Mattson MP (1997). Amyloid *β*-peptide impairs glucose transport in hippocampal and cortical neurons: involvement of membrane lipid peroxidation. *Journal of Neuroscience*.

[71] Markesbery WR (1997). Oxidative stress hypothesis in Alzheimer’s disease. *Free Radical Biology and Medicine*.

[72] Yatin SM, Varadarajan S, Link CD, Butterfield DA (1999). In vitro and in vivo oxidative stress associated with Alzheimer's amyloid beta-peptide (1–42). *Neurobiol Aging*.

[73] Butterfield DA, Lauderback CM (2002). Lipid peroxidation and protein oxidation in Alzheimer’s disease brain: potential causes and consequences involving amyloid *β*-peptide-associated free radical oxidative stress. *Free Radical Biology and Medicine*.

[74] Mohmmad Abdul H, Wenk GL, Gramling M, Hauss-Wegrzyniak B, Butterfield DA (2004). APP and PS-1 mutations induce brain oxidative stress independent of dietary cholesterol: implications for Alzheimer’s disease. *Neuroscience Letters*.

[75] Sanchez-Mejia RO, Newman JW, Toh S (2008). Phospholipase A2 reduction ameliorates cognitive deficits in a mouse model of Alzheimer’s disease. *Nature Neuroscience*.

[76] Sanchez-Mejia RO, Mucke L (2010). Phospholipase A2 and arachidonic acid in Alzheimer’s disease. *Biochimica et Biophysica Acta*.

