# Supplementary material for: Plasmalogens Inhibit APP Processing by Directly Affecting γ-Secretase Activity in Alzheimer's Disease
Source: ScientificWorldJournal. 2012 Apr 1;2012:141240. doi: 10.1100/2012/141240 (PMC3322458; doi:10.1100/2012/141240)

Supplement Rothhaar *et al*

Fig. S1
Cytotoxicity after lipid incubation

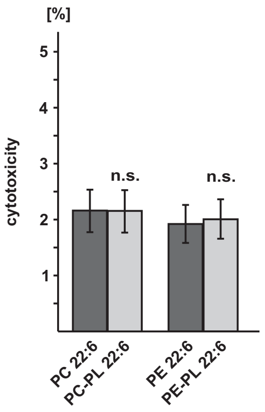

Fig. S2
Control of specifity in α-secretase assay

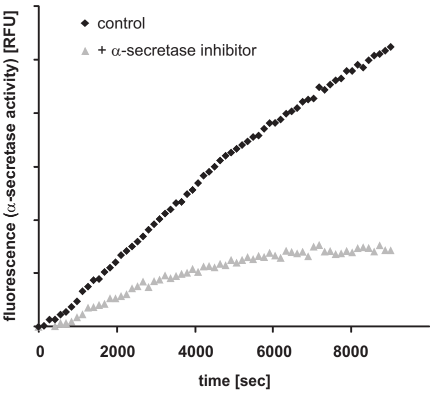

Fig. S3
Control of specifity in β-secretase assay

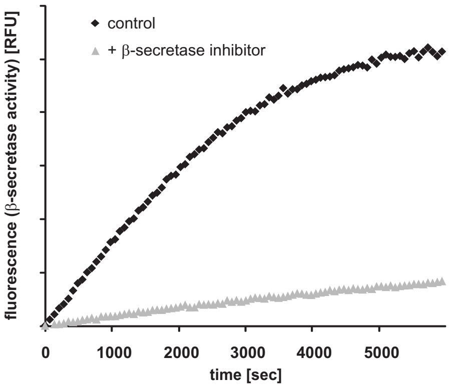

Fig. S4
Control of specifity in γ-secretase assay

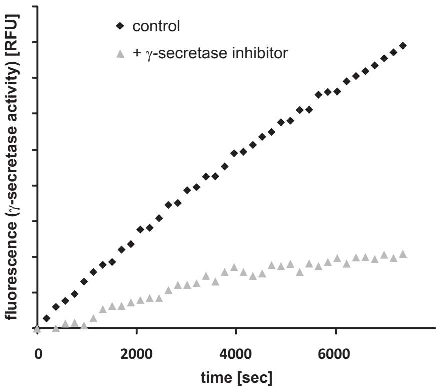

Supplement: Supplementary file 1 — The supplemental information contains the cytotoxicity measurements after PC-PL and PE-PL incubation. Further, specificity controls of secretase assays for α-, β- and γ-secretase were presented. All methods used in the supplemental information are described in the Material and Method section within the manuscript. [file 141240.f1.pdf]
